# Determinants of tourists’ length of stay

**DOI:** 10.1371/journal.pone.0259709

**Published:** 2021-12-07

**Authors:** Ove Oklevik, Grzegorz Kwiatkowski, Ewa Malchrowicz-Mośko, Luiza Ossowska, Dorota Janiszewska

**Affiliations:** 1 Department of Business Administration, Western Norway University of Applied Sciences, Sogndal, Norway; 2 Department of Marketing, Management and Finance Institute, WSB University in Gdansk, Gdansk, Poland; 3 Department of Economics, Faculty of Economic Sciences, Koszalin University of Technology, Koszalin, Poland; 4 Department of Sports Tourism, Faculty of Sport Science, Eugeniusz Piasecki University of Physical Education in Poznan; Poznan, Poland; University of Thessaly, GREECE

## Abstract

This paper aims to identify the determinants of the length of stay (LoS) of international tourists in Norway. The paper reassesses the standard assumption related to tourists’ LoS; it refers to the travel industry’s current trends, and it postulates a more sustainable approach to analyzing tourists’ LoS at the destination level. The paper concludes with a series of recommendations. The data for this study were collected during 153 data collection days and among 5,300 travelers in Norway. The determinants of LoS were analyzed by means of an ordinary least squares (OLS) regression. The results indicate that tourists’ LoS is positively related to their age, interests (nature-based tourists), origin (German, Dutch tourists) and mode of travel organization (package tourists). A negative and significant effect on tourists’ LoS was found for tourists’ interests (urban-based tourists), spending, and origin (home market, long-haul tourists). No significant results were revealed for two covariates, namely, gender and repeat visitation.

## 1. Introduction

For what length of time do tourists stay at a destination? What factors determine their length of stay (LoS)? Is it possible to define an objective group of LoS determinants? The answers to these questions are of crucial importance for understanding how to create more sustainable tourism development. The study argues that more extended stays are more sustainable as the potential effects of tourism interaction with local communities and business entities are less intense and hectic. The longer stays allow tourists to spread around the region and visit secondary tourist attractions and places. This can, in turn, benefit economically more local inhabitants, particularly those who live outside tourists’ primary concretion.

Furthermore, such information can serve as a proxy for calculating the direct economic impact of visitors spending [1] and drawing policy recommendations for accommodation and transport companies [2–4]. Indeed, tourists’ LoS has significant consequences for the destination’s economy, as it directly affects tourists’ demand within, *among other things*, the hotel and foodservice sector [3, 5–8]. Therefore, destination managers are faced with how to attract visitors who stay longer at the destination. Thus, the need to better understand what influences tourists’ LoS is evident. For instance, if increasing age is associated with a more extended stay, destination products/services and respective marketing efforts should be tailored to attract an elderly audience. Within the past 30 years, hotels have developed systems of revenue management that are based upon similar systems developed by the airline industry. The purpose of the revenue management systems for hotels is to maximize revenues and, ultimately, profits by using a variety of tools and strategies to manage space, time, and revenue [1]. A key variable in those systems pertains to the information about the visitor’s length of stay, as it defines different segments of customers for marketing tailoring.

To date, a broad strand of literature has revealed a relation between tourists’ LoS and consumption patterns [5, 6]. While more extended stays usually correspond to higher total expenditures, shorter stays tend to generate higher per day expenses [9–12]. However, the review of existing studies indicates that it is not ultimately clear what determines visitors’ LoS *per se* or which type of stay (shorter vs. longer) provides more sustainable outcomes for the host and why this is the case [13, 14]. This study aims to fill in this research gap.

Against this background, the main aim of this paper is to identify the determinants of the length of stay (LoS) of international tourists in Norway. The results indicate that a very large group of determinants influences the LoS in many directions, and it is complex task to identify an objective group. This can be caused by several context- and time-related variables, that can drive results in particular tourists destinations. Financial and economic factors most objectively and directly affect the LoS. This may be due to the hard and measurable nature of this variable. The remaining determinants refer to more subjective phenomena; hence, it is more difficult to define their relationship with LoS. The determinants of repeat visits or home market tourism result from the individual approach of the tourists. These determinants are based on tourists’ individual experiences and expectations. Generally, age (older), budget and natural spaces are more important for longer stays, while shorter stays are associated with age (younger), prices and urban spaces.

The remainder of this paper is organized as follows. Section 2 reviews the existing research on LoS. Section 3 introduces methods and results. Section 4 includes discussion, and section 5 concludes the paper.

## 2. Literature review

LoS is an essential parameter for tourism destination management. However, research by Jacobsen et al. [15] indicates that on a global scale, LoS is declining. The trend to undertake more short trips throughout the year has meant that the tourist industry has started to show greater interest in attracting the tourist segments that engage in prolonged stays, as these segments are very profitable [14].

LoS has direct implications on the social, economic and environmental viability of host destinations. Tourists’ LoS impacts modes of infrastructure and resource use. As indicted by Gössling et al. [16], shorter stays cause more intense demand for transport infrastructure, as greater tourist volumes need additional airport capacity or other transport infrastructure. Shorter stays may force tourists to focus exclusively on must-see attractions, thereby making other regions/attractions somewhat forgotten [16]. This unbalanced share of tourists is a use of geographical space that may lead to overtourism in one area and stagnation in other areas. In contrast, tourists who stay longer may visit a greater number of potentially smaller businesses in more peripheral locations. They are also likely to develop more complex destination images. Furthermore, due to their longer stays, the social and environmental impacts (costs) of such tourists are less intensive, spread over a longer period of time, and often distributed across larger areas. Therefore, tourists with a longer LoS may create fewer regions with a very high concertation of tourists (’overtourism hot spots’). Thus, to better optimize the tourists’ visits in the host areas, there is a clear-cut need to understand the drivers of the longer stays of tourists. In the following, a review of some international contributions is given, and based on this review, some research gaps and avenues for further research are outlined.

The determinants of LoS have been analyzed by tourism researchers in Europe and in tourist destinations outside of the Old Continent. Such investigations have been conducted for not only regions that are famous for mass and beach tourism but also those regions—such as cultural or sporting tourism areas—that offer a more demanding form of leisure time spending. Analyses have been conducted for different types of tourists, e.g., those visiting friends and relatives or senior travelers. In the following table, for the studies conducted to date, an outline of some critical contributions is given to show the studies’ tremendous geographical diversity, main findings and methodological approaches (Table 1).

**Table 1:** Overview of the previous studies on LoS.

Authors	Location	Sample	Methods	Positively related covariates	Negatively related covariates
Gokovali et al. (2007)	Bodrum, Turkey	672	Cox and Weibull models	nationality (Russian); income; international tourist experience; nonpackaged vacations; reservations in advance; past visits; attractiveness of natural and cultural environment; standard of nightlife and entertainment; overall attractiveness and image of Turkey	nationality (British); level of education; average daily spending; number of vacations taken abroad per year; type of vacation (all-inclusive); type of accommodation (yacht); level of local hospitality
Martínez-Garcia and Raya (2008)	Catalonia, Spain	990	Cox survival models, log-logistic	occupation; reason for visiting the selected destination	nationality (UK, Ireland, Holland and Belgium); age (>40); education; visitation during the high season
Gomes de Menezes et al. (2008)	Azores, Portugal	400	Log-logistics and Cox model	nationality (Portuguese tourists from the mainland); education (university degree); travel party structure (with other adults); destination image (cultural heritage)	azorean ascendancy; motivation (visiting friends and business); repeat visitation; charter flight travel; number of islands visited; sustainable practices; destination image (weather and ultra-periphery areas)
Barros et al. (2008)	Latin America	442	Cox model, Weibull model, logistic model c	budget; destination attributes (nature, culture, climate, gastronomy); social class; frequency of travel (frequent traveler)	destination attributes (ethnicity, exotic, security); age; party size; importance of information (brochure)
Barros and Machado (2010)	Madeira; Portugal	346	Weibull model	repeat visitation; age (older tourists); gender (male); education (more educated); nationality (German); casino visits; visits for island flora and fauna exploration); quality of the accommodations	nationality (British, Dutch, French); expenditures.
Barros et al. (2010)	Algarve, Portugal	593	Cox model, Weibull model	nationality (British, German, Scandinavian, French); education; daytime golf playing; motivation; accommodation type; destination attributes (climate, events, hospitality)	destination attribute (beach)
Raya (2012)	Barcelona, Spain	346	Weibull model, log-logistic; log-normal	evaluation of the destination; expenditure; accommodation type; party size and structure	-
Peypoch et al. (2012)	Madagascar	615	Fractional polynomial model	income; age (older); gender (male); education (higher); destination attributes (nature, sea and security)	travel costs; destination attributes (gastronomy, lifestyle).
Salmasi et al. (2012)	Italy	11,094	Quantile regression	income; party size; marital status (single, widowed); destination type (touristic); transportation mode (car rental, plane, ship, train); accommodation type (village, camping, rented house, multiproperty, free house)	season (1st, 2nd and 4th quarter); year (2006, 2008); price of touristic service; age (< 65); destination location (north-west, central); destination type (mountain, lake, countryside rural, cultural, study/sport); accommodation type (other)
Thrane (2012)	Norway	539	OLS, Log-normal, Lo-logistic, Weibull model	foreign trips; trips booked on the internet; trips taken in July; charter tours; planning time for a trip; motivation (escape motive)	expenditures per day; time constraints
Thrane and Farstad (2012)	Norway	2,895	OLS	nationality (Danish, British, Dutch, German, Other European); age; number of previous visits in Norway, number of places visited; satisfaction	expenditures per day; package tours
Brida et al. (2013)	Italy	724	Binominal model	income (< 20,000); attraction (Otzi museum); bad weather; age (>60)	nationality (Italians, Netherlands); age (<30); employment status
Alén et al. (2014)	Spain	358	Binominal model	age; visits to friends or relatives); destination’s climate; independent travel; accommodation type (apartment, second residence); activities (shopping, organized day trips, physical activities)	-
Kruger and Saayman (2014)	Kruger National Park, South Africa	175 (the northern region) and 235 (the southern region)	Poisson regression model	northern region: total spending; loyalty card; decision to visit made: long in advance; lion and leopard as ’must-see’ big five animals southern region: decision to visit made: long in advance; motive ’escape’; total spending; loyalty card	northern region: travelling from Gauteng; money for conservation southern region: Afrikaans; mode of transport: sedan; travelling with a larger group
Santos et al. (2014)	Brazil	309,000	Weibull model	travel purpose (sun and sea, friends and relatives); individual tourist trips; type of tourist travel (international trips by air); accommodation type (friends and relatives, rented dwellings, own dwellings); summer season travel; type of destination (coastal)	gender (men); age; education (graduate and postgraduate); place of origin (South Americans); visiting more than one destination; travel purpose (business); accommodation type (hotels); party size; first time visitor trips; expenditures
Prebensen et al. (2015)	Northern Norway	986	OLS	time spent in N. Norway worthwhile; time spent at attraction worthwhile; ruggedness/ sincerity; socialization; maintenance/ functional value; intercept	gender (female); N. Norway represents value for money; self-improvement; risk probability;
Rodríguez et al. (2018)	Santiago de Compostela, Spain	10,044	Probit and truncated regression, Heckman model	motivation (business, congress); transport; principal; distance; promotion; attractiveness	gender, occupation (entrepreneur, employee, retired, student), season (low), organization, group; crisis; jubilee; motivation (religion)
Wang et al. (2018)	Macao, China	5,855	OLS	repeat visit, information source (word-of-mouth information, magazines, the Internet, television), destination status (the egress destination), transportation (airplane), companions (traveling alone, young companions—children)	-
Montaño, et al (2019)	Spain	_	General autoregressive, distributed lag model	gross data from airports; arrival and departure numbers; lag of 32, 65 and 95 days	-
Soler et al. (2020)	Malaga, Spain	674	Binominal model	type of accommodation (friend’ s/family house, second home, rented house, apartment); transportation type; dependent children (yes); age; gender (female).	traveling in a group; material status (divorced); income.
Atsis et al. (2020)	Istanbul, Turkey	414	Truncated Poisson regression model	first visit; previous length; historical attributes; cultural attributes; wellness shopping	hotel medium; hotel low; before in cultural; intangible attributes; business
Bavik et al. (2021)	Macau, China	847	Poisson regression model	availability of time, package; reservation time; repeat times; recommendation; services; environment; gastronomy; children; distance; image; outdoor; weather; events; shopping	spending; companion; hospitality; nightlife; accommodation; safety; beaches

Through a survival analysis that used data obtained by conducting a questionnaire survey, Gokovali et al. [4] investigated the determinants of LoS in Bodrum (Turkey) during the summer season. The authors checked almost 40 variables, and 16 of the variables were found to be significantly associated with tourists’ decisions about their LoS. The most critical factors positively related to LoS were annual household income, experience as an international tourist, past visits to the destination, the attractiveness of the natural and cultural environment, the standard of the nightlife and entertainment, and the overall beauty and image of Turkey. The factors negatively related to LoS were tourists’ education, the type of vacation, the type of accommodation, and the local hospitality level. Atsiz et al. [17] also conducted a study on the LoS in Turkey, but the purpose of the analysis was cultural tourism in Istanbul. The research was divided into two main stages. In the first stage, the characteristics that increase or decrease the probability of one might tourism were investigated. Then, the determinants of the length of stay were investigated for tourists staying longer in the destination. According to the results, the LoS was positively influenced by: first visit, previous length, historical attributes, cultural attributes and wellness shopping. While the negatively affected: hotel medium, hotel low, before in cultural, intangible attributes and business. The authors found a positive impact of cultural attributes on tourists’ LoS, which was crucial for the conducted research.

Martinez-Garcia and Raya [18] analyzed LoS for low-cost tourism in Catalonia (Spain). They tried to explain to what extent the tourists’ characteristics, those of the journey and the stay, and those of the tourist destination itself were significant in determining the length of a trip. The authors estimated an econometric duration model and found that for explaining the observed differences in stay duration, the effects of time restrictions, the tourist’s spending capacity, prices, and the differences between urban and *sea-sun-sand* destinations seemed relevant. According to Martinez-Garcia and Raya [18], aspects such as occupation and reasons for visiting tourist regions are more important than age or nationality in regard to making decisions about vacation duration. Rodriguez et al. [19] also conducted research in Spain, in Santiago de Compostela. Noteworthy is the large sample and the length of the research (2005–2012). In a complex analysis process, the authors analyzed different variants. The results confirm the influence of most variables in terms of personal characteristics, travel and destination on the LoS.

Alén et al. [14] indicated that the determinant factors of senior tourists’ LoS in Spain were the following: age, travel purpose, climate, type of accommodation, group size, trip type and the activities carried out at the destination. Similarly, Gomes de Menezes et al. [20] examined the determinants of tourists’ LoS in Portugal (Azores Islands). Among the most critical factors, the authors distinguished repeat visitation rate (destination loyalty), type of flight and destination image (weather and ultraperiphery areas). They also claimed that for tourists who stayed longer in the Azores, natural heritage was a more attractive aspect than cultural heritage.

To examine the LoS for international triathlon participants in the Barcelona region, Raya [21] investigated sports tourists’ behaviors. The study underlined that the economic impact of events on tourism depends not only on the number of participants but also, among other things, on the LoS at the tourist destination. Raya analyzed the factors determining the LoS of triathletes and suggested that satisfaction with the destination, the resident status of the participant (foreign or domestic), the type of accommodation, the event size, the structure and the participant’s expenditures appear to have a significant influence on the participant’s decision of how long to stay at the sports destination.

Barros et al. [22] were interested in the LoS of golf tourists in the Algarve region (Portugal). They concluded that the LoS is positively related to the following: the nationality, age and level of education of the respondents; the climate; accompanying events; and the local hospitality. According to Barros & Machado [23], who analyzed the factors affecting the length of tourist stay in Madeira, the most important factors were repeated visitation and accommodation quality, while the factor of expenditure amount had less importance.

Brida et al. [24] examined the LoS of cultural tourists in Italy. For this type of tourist, they identified the following as the main determinants that influence the LoS: nationality, age, employment, and the income and costs associated with the journey. The authors emphasize that in terms of age groups and employment status, the LoS is shorter in the group under 30 years of age. This is due to both the lack of free time in this group and the lower level of income. Salmasi et al. [25] investigated tourist behaviors in Italy and found that the positively related aspects to length of stay were income, marital status (single, widowed), transport mode, and accommodation type, while the negatively related aspects were holiday season travel, the prices of the tourist services, the destination locations, the destination type and the accommodation type.

Peypoch et al. [26] examined Madagascar’s tourist situation and found that for LoS, the positively related aspects were income, age, education and destination attributes (nature, security, etc.), while the negatively related aspects included travel costs. Barros et al. [27] tried to explain the determinants of the LoS in Latin American tourist destinations. They found that the LoS functions more as a determinant of destination demand than a demand constraint and is mostly explained by travel costs, the effect of which is moderated by the destination’s perceived characteristics, publicity, and the tourist’s sociodemographic profile. Barros et al. [27] found that the factors positively related to staying duration are budget, certain destination attributes (nature, culture, climate, gastronomy, etc.), and the social class represented by a traveler. They found that the factors negatively associated with staying duration are certain destination attributes (e.g., ethnicity), the age of the traveler, and available information (brochures, etc.).

Thrane [7] analyzed the LoS of international summer visitors in Norway, and the study results showed that nationality explains many of the differences in LoS among international visitors in Norway. The results also highlighted how international visitors’ age, spending patterns and other trip-related characteristics are associated with LoS. Thrane and Farstad conducted another study in Norway in 2012 [28]. The authors indicated that the number of previous visits to Norway, the number of places visited and satisfaction are positively related to LoS, while expenditures per day are negatively associated. Subsequent studies in Norway—in the northern part—were carried out by Prebensen et al. [29]. Due to the specificity of the region, the authors concluded that tourists are looking for authentic and natural experiences, while tourists with higher incomes may not find the luxury offers they expect. Thus, the authors observed the effect of motivation of motivation, destination perception and experience value.

Santos et al. [30] analyzed LoS factors in Brazil. The research was aimed at understanding tourists’ behaviors and predicting their length of stay according to relevant variables. Soler et al. [31] referred to the model proposed by Alén et al. [14]. In addition to common factors, LoS research has also investigated the effect of the climate index.

Wang et al. [32] conducted the LoS study in Macau, a major gaming destination in Asia. Authors verified the superiority of models with the log-transformed LoS. Based on the research, the authors identified the features affecting the LoS: repeatability, information source, means of transport and destination status. Bavik et al. [33] also analyzed the LoS of tourists in Macau. They examined over 20 different features. The authors drew attention to destination attributes as the hypothesis of their positive impact on the LoS was only partially confirmed (services, environment, gastronomy, children, distance, image, outdoor, weather, events, shopping). Some of the destination attributes turned out to be negatively correlated with the LoS (hospitality, nightlife, accommodation, safety, beaches). As the authors point out, the results were undoubtedly influenced by the fact that only Chinese tourists were researched, for whom this destination is associated with shorter trips related to entertainment and gambling.

An interesting case in the field of natural based tourism was described by Kruger and Saayman [34]. The authors conducted research in the Kruger National Park—in the northern and southern region. The results vary considerably despite the similar destination and area combined into a national park. The authors emphasize that LoS determinants require a regional approach.

In addition, Malchrowicz-Mośko & Rozmiarek [35] examined sports tourists’ LoS during the European Swimming Championships in Poland. They indicated that if the organizers of sporting events would prepare unique cultural and tourist offerings in collaboration with the local tourist authorities, foreign visitors would prefer to stay longer in Poznan.

The most common method to explain LoS is survey combined with regression analysis (Table 1). However, some new approaches exist. Montano et al. [36] have recently developed another tool for explaining LoS. They used historic airport data of numbers of arrivals and departures, and showed how the use of lagged data (32, 65 and 95 days) could give precise predictions of LoS. This contribute to literature to explain *how* LoS develop over time, but not *why*.

Apart from determinants, research on LoS also includes sustainable tourism. The LoS is regarded as one of the indicators of sustainable tourism development. The environmental effects of the LoS relate to energy consumption, water consumption, waste generation, carbon dioxide emissions, etc. [37]. According to studies on environmental pollution, especially greenhouse gas emissions, as a factor affecting the environment, LoS should be considered together with the average distance traveled by tourists [16]. More but shorter journeys increase the overall amount of transport emissions [38]. Longer stays are better both economically and environmentally. More extended stays may reduce the need for a continuous increase in the number of tourists and may reduce the amount of anthropogenic pressure. Furthermore, longer stays can open up possibilities to activate core tourist attractions and attractions located at the peripheries of tourists’ interests. Such an approach may open numerous options for local community empowerment. Therefore, some studies have argued that it is more critical to seek the optimization rather than the maximization of tourists’ length of stay, as the optimization of the length of stay will result in greater sustainability [16, 38, 39]. The optimization of LoS, instead of the maximization of LoS, is needed because LoS mediates the relationship between environmental pollution and tourism income. The worse the perception of the tourist destination’s beach environment is, the shorter the tourists’ stays are and the less they spend [40].

## 3. Methods and results

To understand the drivers of LoS, a survey of international leisure tourists was conducted in southwestern Norway. Comprising the counties of Hordaland, Rogaland, and Sognog Fjordane, this region has a population of approximately 1.1 million [41] and is internationally branded as ’Fjord Norway’. The most important origination markets include Germany, the Netherlands, France, Denmark, Sweden, the United States, and the United Kingdom [39].

Data were collected between May 25, 2016 and September 15, 2016, for a total of 153 data collection days. Faktum Analyze AS, a company specializing in surveys, interviewed the tourists. Questionnaires were developed in Norwegian and then translated into English and German to adequately capture information from tourists who had arrived by different transport modes. No problems in filling out the questionnaires were encountered by other nationalities. Because no specific probability structure was expected, a nonprobability sampling technique was used. However, the survey days were varied across the weeks of data collection to reduce the level of potential sampling bias (temporally stratified sampling) [42]. The interviews were carried out in six locations, including the ferry terminals in Hella, Lavik and Kristiansand and the airport in Bergen, as well as in the center of Bergen. One location covered two central exit points from the area/Norway, that is, the exit points for passengers waiting for departure at Kristiansand seaport (16 days, a response rate of 66%) and Bergen airport (38 days, a response rate of 43%). To cover visitors exiting in the northern part of the region, questionnaires were handed out to travelers waiting for departure at two ferry landings at Sognefjord (Hella: 29 days, a response rate of 70%; Lavik: 15 days, a response rate of 58%). To avoid responses from travelers residing in Norway, a screening question was included. As the Kristiansand seaport is outside the study region, an additional screening question was used to identify passengers who had visited the counties of Sognog Fjordane, Hordaland and/or Rogaland (which are in the Fjord Norway region). In addition, questionnaires were randomly distributed to foreign vacationers in the Bergen city center (55 days, a response rate of 45%; the low response rate was influenced by frequent occurrences of rain).

The data was collected by Western Norway Research Institute together with their industry partners and there were some strict rules about how to deal with data after collection is done. No name or any information was available which might be use to identify respondents. No youths below 18 have been interviewed. Participation in the study was voluntary and the respondents were informed about the purpose of the study.

In line with earlier research on airport exit surveys [43], the response rates varied between 43–70%. The interviews lasted between 10–15 minutes. In total, 5,283 questionnaires were completed and returned to the interviewers. The questions addressed LoS, participation in 33 types of activities, spending, gender, age, country of residence, tourist type (package tourist or not) and visit frequency (repeat visitors). The respondents were asked about their participation in 33 types of activities and were directed to provide responses in a manner consistent with the official distinction used by Fjord Norway and the Norwegian authorities [44]. This meant that the tourists reported on the type of activities they had participated in instead of the frequency of their participation. Based on a discussion between the authors, the 33 types of activities were categorized into three main activity types. These three categories consisted of culture-based, nature-based and urban-based activities. In the final stage of categorizing, each tourist was defined as either a nature-based, a culture-based, or an urban-based tourist if the number of counted activities in one of the categories exceeded those in the other two. However, tourists who had an equal number of activities in two or more groups or had not participated in any activity were defined as being in the control group.

Table 2 shows the respondents’ distribution by gender, age, and nationality, indicating that approximately half of the respondents were male (50.4%) and approximately half were female (49.6%). The age distribution indicates a large share (24.2%) of younger tourists (25–34 years old). The nationalities included German (26.3%), British (11.0%), and US citizens (10.7%).

**Table 2:** Sample demographics.

**Gender**		
Male	2 555	50.4
Female	2 516	49.6
**Total**	**5 061**	**100.0**
**Age**		
		
18–24	918	18.2
25–34	1 221	24.2
35–44	722	14.3
45–54	844	16.7
55–64	777	15.4
65-	563	11.2
**Total**	**5 045**	**100.0**
**Nationality**		
Nordic countries	389	7.4
Netherlands	378	7.1
Germany	1 389	26.3
UK	581	11.0
US	567	10.7
Asia	327	6.2
Other countries in Europe	1 244	23.7
Other countries	408	7.1
**Total**	**5 283**	**100.0**

**Fig 1:**
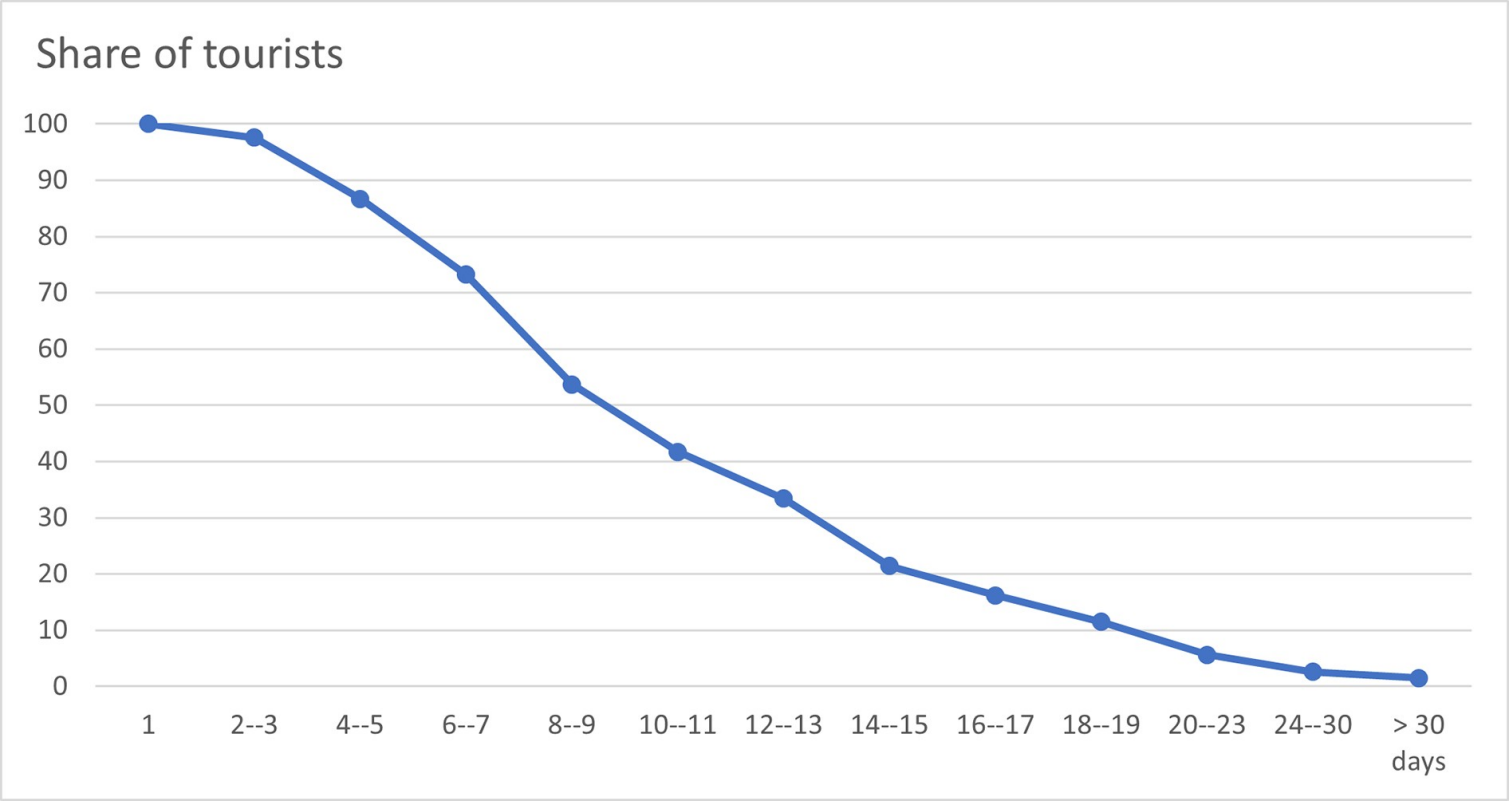
Share of tourists distributed over LoS.

While all the tourists had been in Norway for at least 1 day, 2.4% of the tourists left the region during days 2 and 3, while 97.6% remained in the area on day 3. By day 5, 13,3% of tourists had left the area, while 86,7% still remained. After 7 days, 26,8% of the tourists had left the area. Fig 1 shows the share of tourists who stayed in Fjord Norway over time. The most common travel pattern among international tourists in Western Norway is an LoS of 6–7 days, which was the LoS for 19,7% of the tourists. Furthermore, 13,1% of the respondents stayed for a period of 14–15 days, while 11,1% stayed for 10–11 days.

To further investigate the impact of the independent variables on LoS, an OLS regression model was run. LoS was used as the dependent variable and was measured by the number of days of a stay in Norway. For the independent variables, the model also included three dummy variables, which were used to indicate whether the tourist could be categorized as a mainly nature-based, culture-based or urban-based tourist (here, tourists who were involved in no activities or had the same number of activities across two of the defined categories served as the comparison group). Furthermore, the model used the tourists’ average spending per day as a continuous independent variable and included a dummy variable to denote a package tourist. The latter variable captured tourists who were booked on package tours. Finally, as independent variables, the model included six dummy variables representing Norway’s most important geographical markets [39]: the US, the UK, Asia, the Netherlands, and the Nordic countries. In the OLS model, the group of tourists from all other countries functioned as a control group.

The variables included in the final OLS model are listed and explained in Table 3. The table also includes the expected impact of the variables on LOS. Spending should have a negative impact on LOS, as people usually have a fixed holiday budget. Staying longer means that the budget has to be distributed over more days. The expected impact of repeat visitors on LOS is positive because tourists revisiting a destination have learned about the destination from their previous visit and will be more able to plan activities beforehand. We expect nature-based and culture-based tourists to stay longer at the destination, as they have to travel around the country to fulfill their activities needs, and this method is time consuming. However, the opposite is expected for urban-based tourists, as theirs tourism goals are easier and they need less time costs to reach their target. We expect that older individuals should generate a higher level of LOS as income usually increases with age. Furthermore, a substantial portion of the highly aged population will be retired and in a position to spend more time on the destination. Package tourists are expected to stay longer, as they usually obtain reduced prices through their packages, which might encourage for longer stays. Regarding geographical market impacts, we expect long-distance travelers such as US tourists and Asian tourists to negatively influence LOS because they are traveling by plane and must allocate their time in Europe between several destinations. Additionally, UK tourists might also be at the destination for a short time because they are traveling by plane. On the other hand, more short-distance travelers to the Norwegian market, such as those form the Netherlands and Germany, are expected to have a higher LOS because they tend to travel by car. This means that they have more flexibility and might undertake a larger amount of activities than their air-traveling counterparts. Previous research has shown that people from the Netherlands and Germany are important tourists for Norway because they stay longer than other groups [39]. Finally, tourists traveling from Nordic countries should stay in Norway for a shorter time, as they can travel to the destination on a more frequent basis.

**Table 3:** Variables in the models.

Variable	Description	Expected impact
Dependent variable: LoS	Continuous variable; number of days in Norway.	
Spending	Continuous variable; average spending per visitor and day.	-
Repeat_Visitor	Dummy variable with a value of 1 if the traveler visited the area earlier and a value of 0 otherwise.	+
Nature-based-Tourist	Dummy variable with a value of 1 if the traveler is mostly involved in nature based activities and a value of 0 otherwise.	+
Culture-based-Tourist	Dummy variable with a value of 1 if the traveler is mostly involved in culture based activities and a value of 0 otherwise.	+
Urban-based-Tourist	Dummy variable with a value of 1 if the traveler is mostly involved in urban-based activities and a value of 0 otherwise.	-
Age	Continuous variable; age is measured in years.	+
Gender	Dummy variable with a value of 1 if the traveler is a man and a value of 0 if the traveler is a woman.	+
Package-tourist	Dummy variable with a value of 1 if the tourist has purchased a package trip and a value of 0 otherwise.	+
D_Asia	Dummy variable with a value of 1 if the tourist is from Asia and a value of 0 otherwise.	-
D_Germany	Dummy variable with a value of 1 if the tourist is from Germany and a value of 0 otherwise.	+
D_US	Dummy variable with a value of 1 if the tourist is from US and a value of 0 otherwise.	-
D_UK	Dummy variable with a value of 1 if the tourist is from the UK and a value of 0 otherwise.	-
D_Netherlands	Dummy variable with a value of 1 if the tourist is from the Netherlands and a value of 0 otherwise.	+
D_Homemarket	Dummy variable with a value 1 if the tourist is from a home market (Nordic countries and a value of 0 otherwise.	-
Interaction_D_Germany_*_Urban-Based_	Interaction variable (measured as dummy). 1 if the respondent is a German and Urban based classified tourist. 0 otherwise.	

The results from the OLS are reported in Table 4, which shows the unstandardized regression coefficients (beta values) and adjusted R2 values. The inspection of the model evaluation (adjusted R2) shows a good model fit, with an adjusted R2 of 0.22. Some scholars argue that in tourism studies estimating factors influencing LoS, survival models should be used [4, 20, 27]. Survival models originated in studies about the labor market [45]. However, empirical evidence show that the results are the same independent of type of model. In the study of Thrane [7] shows that the OLS regression model describes the impact of independent variables on length of stay at least as effectively as survival models. Thrane [7] argue that OLS is superior compared to survival models because it allows negative impact of independent variables on the dependent variable, while survival models do not. Further, Thrane [7] also argue that "In line with the principle of parsimony it is concluded that future studies on tourists’ length of stay should abandon survival models". In our study, since we expect there to be negative impacts on LoS for some of our independent variables, we follow the advice of Thrane [7] and use OLS as estimation method.

**Table 4:** Unstandardized regression coefficients.

Variable	Model	t-values
Intercept	4.36***	5.14
Spending	-0.01***	-7.98
Nature-based-Tourist	1.34***	4.31
Culture-based-Tourist	0.93^n^***	2.81
Urban-based-Tourist	-0.96***	-2.35
Age	0.07***	8.32
Package-tourist	2.45***	6.89
D_Asia	-3.90***	-5.64
D_Germany	156***	3.04
D_US	-2.27***	-4.97
D_UK	-3.58***	-8.32
D_Netherlands	3.00***	5.73
D_Homemarket	-4.13***	-7.87
Adj R2	0.23	
Interaction_D_Germany*Urban-Based_	1.75***	2.97

**Note:** Dependent variable: LoS; n.s. denotes not significant

* denotes significant at the 10% level

** denotes significant at the 5% level

***denotes significant at the 1% level.

In the first step of developing our model, we included all the variables in Table 3, + some additional ones like gender, repeat vistor and aseveral interaction variables between all the country measures and the three categories of tourist mentioned (nature_bsaed, culture_based and Urban_ based). In the next step, we excluded the non-significant independent variables. This move gave us a final list of independent variables as in Table 4. Only one significant interaction varable remained, the interaction term between being a German and Urban based tourist.

The results show several direct effects of the independent variables on LoS. Regarding activities, being a nature-based tourist has a positive and significant impact on LoS (β = 1.34, p<0.01), while being an urban-based tourist has a negative impact on LoS (β = -0.96, p<0.01). Furthermore, being a culture-based tourist had an impact on LoS (β = 0.93, p<0.01). The results is mixed. While being a culture-based or a nature-based tourist contributes to a longer stay in Norway. But being a urban-based tourist contributes to a shorter stay. Our first model showed that compared to being a first-time visitor, being a repeat visitor does not have any impact on LoS. Further, the results show that age has a positive impact on LoS (β = 0.07, p<0.01), which means that older tourists stay longer in the area than their younger counterparts. However, no gender effect exists. Furthermore, package tourists have a positive impact on LoS (β = 2.45, p<0.01). Regarding the country of origin, the dummy variables for the most important markets for the tourism industry in Norway were included. Here, there are several different effects on LoS. Being a German or Dutch tourist has a positive and significant impact on LoS (β_German_ = 1.57, p<0.01, and β_Netherlands_ = 3.01, p<0.01). However, being a tourist from the US, the UK, Asian or the Nordic home market negatively impacts LoS (β_US_ = -2.27, p<0.01, β_Asia_ = -3.90, p<0.01, β_UK_ = -3.58, p<0.01, and β_Homemarket_ = -4.14, p<0.01). One significant interaction effect occurred, the impact of being a German urban-based tourist, had a significant impact on LoS β_Geramny_*_urban-based_ = 1.75, p<0.01. The results appeared further as expected. The peak of the nature-based activity season is between June and the end of August. This timing might have influenced and biased the results. Surprisingly, repeat visitors did not have any impact on LOS.

## 4. Discussion

Referring to previous research results, it is difficult to indicate objective covariates that are positively or negatively correlated with LoS. Substantially, no feature turned out to be only positively or only negatively related to LoS. Researchers emphasize the need for an individual approach to LoS determinants—in relation to the group of tourists or in relation to the destination [32–34].

According to the presented research, some tourist features have a positive effect on LoS, some features have a negative effect, and others have no impact. It is worth adding that in other studies, some results differ from those presented, which could have been influenced by both the sample size and the studied destination.

Economic and financial criteria, such as income and expenditure, seem to be the most objective. Generally, income and budget are positively related to the LoS [5, 25–27], while expenditures, price and cost are negatively related [4, 7, 23, 25, 26, 28, 30, 33]. A holiday budget usually has a predetermined size, and longer stays mean that this budget has to be divided across more days.

Other covariates are characterized by a much greater variation in previous research. Most research refers to gender and age; however, the results of the influence of these features on LoS are not clear. Generally, older tourists’ age has a positive effect on their LoS [5, 14, 23, 26, 28, 31]. These results are similar to our findings. Commonly, older people, especially retirees, have more time at their disposal. They also usually prefer a more peaceful vacation and thus often stay within the same tourist destination.

According to our results, gender has no impact on LoS. Previous research has indicated a gender relation with the LoS, i.e., male positively related [23, 26], female positively related [31] or male negatively related [30], female negatively related [29]. On the basis of the various research results, it is difficult to generalize impacts in this aspect.

Our findings confirm that nature-based tourism is positively related to LoS [4, 26, 27, 29, 30]. Spending leisure time in nature usually takes more days than do trips to the city, which, in turn, are very popular for weekends. This is also confirmed by our results, according to which urban-based tourism is negatively related to LoS. Interestingly, according to our results, culture-based tourism does not have an impact on LoS, which is in contrast to some previous results in which culture was included among the factors positively related to LoS [4, 17, 20, 27].

The results vary in terms of the nationality of tourists. This factor is a feature that is analyzed in many studies, and it is difficult to find a clear direction; however, some nationalities have been surveyed more than others. According to our findings and those of other research [22, 23, 28], being a German is positively related to LoS. German tourists like to travel, especially when they are retired and when they have time to pursue their travel passions.

Our findings confirm that UK tourists prefer shorter stays, which is similar to other results [4, 18, 23]. UK tourists choose weekend trips more often. Therefore, they travel more often during the year but for shorter periods. It is worth adding that there are also examples of studies in the literature that have found that the UK nationality is positively related to LoS [22, 28].

In our research, Dutch tourists are positively related to LoS, which is similar to other research that has been conducted in Norway. Interestingly, in destinations considered more attractive to tourists, the Dutch nationality is negatively correlated with LoS, i.e., in Italy [5], Spain [18] and Portugal [23]. These destinations are more often chosen by Dutch tourists for shorter stays.

Our findings confirm that home market tourism is negatively related to LoS [30], although some previous results have indicated a positive relation [20]. Generally, the aspect of distance may be viewed different in this respect, as well as the possibility of traveling. In a positively related study regarding this relation, the Azores were analyzed, and Portuguese tourists traveled from the mainland.

No unequivocal result was obtained regarding the factor of repeat visits. According to our findings, such visits have no impact on the LoS; however, other research has shown that this relation can be both positive [4, 28, 32] and negative [20] and that first-time visitor trips can be negatively related to LoS [30]. The will to return is an individual decision of each tourist based on his or her previous experiences. This subjective nature makes it difficult to make generalizations regarding this aspect.

In our results, package tourism has a positive relation with the LoS, which is similar to the research conducted in Turkey [4] but in opposition to the other research that has been carried out in Norway [28]. Generally, organized tourism should be conducive to LoS; in addition to week-long or several-week trips, such tourism also includes weekend trips. A lot depends on the tourist destination and the expectations of the tourists.

The authors’ observations regarding the relationship between employment and the LoS are interesting [19, 24]. Employment means that people have less time but receive income. Younger groups in particular tend to stay shorter, which is influenced by less free time and lower income. Retired people, on the other hand, are in a different situation—they have free time and receive income, hence older tourists are willing to go on longer trips. Unemployment is negatively related to the LoS.

It should also be emphasized that the diversity of the research results of various authors, including our results, indicate the great importance of the destination and target group for LoS. In practical terms, the importance of diversifying tourist offers should be emphasized, including, among others, taking into account additional activities and attractions for tourists. Such practices may help to optimize the LoS, thus meeting the expectations of both tourists and entrepreneurs. As a result of the conducted research, various characteristics of tourists influence the length of stay in different ways. Therefore, it is worth knowing who visits a given destination to best match the offers extended to specific customer groups. From a managerial perspective, the study should be used as a proxy for more targeted extended stay policies, which can contribute to better sustainability in host areas. One practical outcome of the study is that the Norwegian travel and tourism authorities, as well as Fjord Norway and other destination companies, should differentiate their marketing strategy toward the German market. With especially emphasize on the urban potential of the region, as this might target this segment’s interest.

Even if the study give some important contributions to the LoS literature, it also have some limitations. The first limitation is that demographic distribution in the survey (for example the nation distribution) cannot be compared to national arrival statistics, as this survey focuses on leisure tourists, while national data includes leisure and business travelers. The study is not representative at national level for Norway. However, as the survey consists of a rather big sample (5283 responses), it should be representative for the tourist population in Western Norway area for the given period the sampling took place (end of May until midst September). The second limitation is that the study doesn’t control for spillover effect. If a destination is surrounded by many other tourist places, they could split their time between different destination [46, 47]. For example, by traveling from eastern Norway to western Norway and vice versa, which in turn might have influenced the results. However, in order to reduce the impact of this potential limitation, one adjustment was planned. One of the locations where the interviews took place, is located outside the region (Kristansand). There a screening question was asked initially, if they had been in Western Norway or not. Therefore, travelers from Eastern Norway were excluded.

## 5. Conclusions

LoS is a very complex, multifaceted problem that requires further research and analysis. Precise determinants of LoS in general are virtually impossible due to the large diversity of groups of tourists and destinations. Furthermore, LoS research can bring about benefits in terms of practical guidance for entrepreneurs, as well as in the dimension of environmental protection, which is becoming a necessity.

The difficulties in finding stable patterns in LoS criteria show the complexity of the problem and that approaching the issue from the perspective of only one feature does not make sense. It is better to consider LoS in the context of a combination of features, taking into account a given tourist area. Understanding LoS requires an individual approach.

To answer the assumed research questions, a very large group of determinants influences the LoS in many directions, and it is hard to define an objective group. Financial and economic factors most objectively and directly affect the LoS. This may be due to the hard and measurable nature of this variable. The remaining determinants refer to more subjective phenomena; hence, it is more difficult to define their relationship with LoS. The determinants of repeat visits or home market tourism result from the individual approach of the tourists. These determinants are based on tourists’ individual experiences and expectations. Generally, age (older), budget and natural spaces are more important for longer stays, while shorter stays are associated with age (younger), prices and urban spaces.

## Supporting information

S1 Appendix. Activities and classifications (DOCX)

S1 Data (SAV)

## References

[1] Wilson RH. Minimum Length-of-Stay Requirements as part of Hotel Revenue Management Systems: Are They Legal? Journal of Hospitality Financial Management. 2001;9 (1):45–54.

[2] Alegre J, Llorenç P. Microeconomic Determinants of the Duration of Stay of Tourists. In *Advances in Modern Tourism Research*, Matias Á, Nijkamp P, Neto P, Eds.; Physica-Verlag HD, 2007; pp. 181–206.

[3] Alegre J, Pou L. The length of stay in the demand for tourism. Tourism Manag. 2006;27(6):1343–1355.

[4] Gokovali U, Bahar O, Kozak M. Determinants of length of stay: A practical use of survival analysis. Tourism Manage. 2007;28(3):736–746.

[5] Brida JG, Scuderi R. Determinants of tourist expenditure: A review of microeconometric models. Tour. Manage. Perspect. 2013;6(0):28–40.

[6] Marcussen CH. Determinants of tourist spending in cross-sectional studies and at Danish destinations. Tourism Econ. 2011;17(4):833–855.

[7] Ch Thrane. Analyzing tourists’ length of stay at destinations with survival models: A constructive critique based on a case study. Tourism Manage. 2012;33(1):126–132.

[8] Archer BH & Shea S. Length of stay problems in tourist research. J. Travel Res. 1975;13(3):8.

[9] Cannon TF, Ford J. Relationship of demographic and trip characteristics to visitor spending: an analysis of sports travel visitors across time. Tourism Econ. 2002;8(3):263–271.

[10] Downward P, Lumsdon L. The demand for day-visits: an analysis of visitor spending. Tourism Econ. 2000;6(3):251–261.

[11] Downward P, Lumsdon L, Weston R. Visitor Expenditure: The Case of Cycle Recreation and Tourism. Journal of Sport & Tourism. 2009;14(1):25–42.

[12] Kastenholz E. Analysing determinants of visitor spending for the rural tourist market in North Portugal. Tourism Econ. 2005;11(4):555–569.

[13] Alegre J, Mateo S, Pou L. A latent class approach to tourists’ length of stay. Tourism Manage. 2011;32(3):555–563.

[14] Alén E, Nicolau JL, Losada N, Domínguez T. Determinant factors of senior tourists’ length of stay. Ann. Tourism Res. 2014;49(0):19–32.

[15] Jacobsen J, Gossling S, Dybedal P, Skogheim TS. Exploring length of stay: International tourism in south-western Norway, J. Hosp. and Tour. Manag. 2018;35:29–35.

[16] Gössling S, Scott D, Hall CM. Global trends in length of stay: implications for destination management and climate change. J. Sustain. Tour. 2018;26(12):2087–2101.

[17] Atsız O., Leoni V., & Akova O. Determinants of tourists’ length of stay in cultural destination: one-night vs longer stays. Journal of Hospitality and Tourism Insights. 2020; doi: 10.1108/JHTI-07-2020-0126

[18] Martínez-Garcia E, Raya JM. Length of stay for low-cost tourism. Tourism Manage. 2008;29(6):1064–1075.

[19] Rodriguez XA, Martinez-Roget F, Gonzalez-Murias P. Length of stay: Evidence from Santiago de Compostela. Ann. Tourism Res. 2018;68:9–19.

[20] Gomes de Menezes A, Moniz A, Cabral Vieira J. The determinants of length of stay of tourists in the Azores. Tourism Econ. 2008;14(1):205–222.

[21] Raya JM. Length of Stay for Triathlon Participants in the Challenge Maresme-Barcelona: A Survival Approach. J. Sport Soc. Issues. 2012;36(1):88–104.

[22] Barros CP, Butler R, Correia A. The length of stay of golf tourism: A survival analysis. Tourism Manage. 2010;31(1):13–21.

[23] Barros CP, Machado LP. The length of stay in tourism. Ann. Tour. Res. 2010;37(3):692–706.

[24] Brida JG, Meleddu M, Pulina M. Factors influencing length of stay of cultural tourists. Tourism Econ. 2013;19(6):1273–1292.

[25] Salmasi L, Celidoni M, Procidano I. Length of Stay: Price and Income at Different Destinations in Italy. Int. J. Tour. Res. 2012

[26] Peypoch N, Randriamboarison R, Rasoamananjara F, Solonandrasana B. The length of stay of tourists in Madagascar. Tourism Manage. 2012;33(5):1230–1235.

[27] Barros CP, Correia A, Crouch G. Determinants of the Length of Stay in Latin American Tourism Destinations. Tour. Anal. 2008;13(4):329–340.

[28] Ch Thrane, Farstad E. Nationality as a segmentation criterion in tourism research: The case of international tourists’ expenditures while on trips in Norway, Tourism Econ. 2012;18(1):203–217

[29] Prebensen N.K., Altin M., Uysal M. Length of stay: a case of Northern Norway. Scandinavian Journal of Hospitality and Tourism. 2015;15(supl.1):28–47.

[30] Santos G, Ramos V, Rey-Maquieira J. Length of Stay at Multiple Destinations of Tourism Trips in Brazil. J. Travel Res. 2014; doi: 10.1177/0047287514532370

[31] Soler IP, Gemar G, Correia MB. The climate index-length of stay nexus. J. Sustain. Tour. 2020;28(9):1272–1289.

[32] Wang L, Fong D, Law R, Fang B. Length of stay: Its determinants and outcomes. Journal of Travel Research. 2018;57(4):472–482

[33] Bavik A, Correia A, Kozak M. What makes our stay longer or shorter? A study on Macau. Journal of China Tourism Research. 2021;17(2):192–209

[34] Kruger M, Saayman M. The determinants of visitor length of stay at the Kruger National Park. Koedoe. 2014;56(2):1–11.

[35] Malchrowicz-Mośko E, Rozmiarek M. Impact of European Junior Swimming Championships in Poznan on Tourism and City Image in the opinion of Athletes. Studies in Sport Humanities. 2017;22:65–71.

[36] Montaño J, Jaume R, Sanso A. A new method for estimating tourists’ length of stay. Tourism Manage. 2019;75:112–120.

[37] Cero JP, Dubois G. Tourism and Sustainable Development Indicators: The Gap between Theoretical Demands and Practical Achievements. Curr. Issues Tour. 2003;6(1):54–75.

[38] Gössling G, Ring A, Dwyer L, Andersson A.-Ch., Hall CM. Optimizing or maximizing growth? A challenge for sustainable tourism. J. Sustain. Tour. 2016;24(4):527–548.

[39] Oklevik O, Gössling S, Hall CM, Steen Jacobsen JK, Grøtte IP, McCabe S. Overtourism, optimisation, and destination performance indicators: a case study of activities in Fjord Norway. J. Sustain. Tour. 2019; doi: 10.1080/09669582.2018.1533020

[40] Qiang M, Shen M, Xie H. Loss of tourism revenue induced by coastal environmental pollution: a length-of-stay perspective. J. Sustain. Tour. 2020;28(4):550–567.

[41] SSB 2017, https://www.ssb.no/befolkning/artikler-og-publikasjoner/dette-er-norge-2017.

[42] Hurst F. En route surveys. In Ritchie J. R. B. & Goeldner C. R. (Eds.), Travel, tourism, and hospitality research (pp. 453–472). New York, 1994, NY: Wiley.

[43] Rideng A, Christensen P. En route surveys. Scandinavian Journal of Hospitality and Tourism. 2004;4(3):242–258.

[44] Visit Norway–Official travel guide to Norway, https://www.visitnorway.com/

[45] Heckman JJ. Sample selection bias as a specification error. Econometrica. 1979;47(1):153–161.

[46] Zhou B, Yang B, Li H, Qu H. The spillover effect of attractions: Evidence from Eastern China. Tour. Econ. 2016;23:731–743.

[47] Romano A. A study of tourism dynamics in three Italian regions using a nonautonomous integrable Lotka–Volterra model. PLoS One. 2016;11(9), doi: 10.1371/journal.pone.0162559 27661615 PMC5035007

