# Supplementary material for: Determinants of tourists’ length of stay
Source: PLoS One. 2021 Dec 7;16(12):e0259709. doi: 10.1371/journal.pone.0259709 (PMC8653829; doi:10.1371/journal.pone.0259709)
Supplement: S1 Appendix. Activities and classifications — (DOCX) [file pone.0259709.s001.docx]

Appendix 1. Activities and classifications

| Activity | Classification | Share Tourist participating |
| --- | --- | --- |
| Kayaking, canoeing | Nature based activity | 8.8% |
| Boating (sailing, motor boating) | Nature based activity | 14.2% |
| Hiking/trekking  (soft) | Nature based activity | 41.4% |
| Hiking/trekking (hard) | Nature based activity | 20.7% |
| Bicycling (road) | Nature based activity | 8.0% |
| Bicycling (mountain) | Nature based activity | 4.3% |
| Fishing (lake – fresh water) | Nature based activity | 6.1% |
| Fishing (fjord, sea – salt water) | Nature based activity | 8.1% |
| Skiing (downhill) | Nature based activity | 1.5% |
| Skiing (cross-country) | Nature based activity | 1.1% |
| Skiing (ski touring) | Nature based activity | 0.9% |
| Snow-shoeing | Nature based activity | 1.0% |
| Ice skating | Nature based activity | 1.1% |
| Hunting | Nature based activity | 0.9% |
| Horse-related activities | Nature based activity | 1.7% |
| Visiting national parks/Naturum | Nature based activity | 27.5% |
| Sun and beach | Nature based activity | 13.5% |
| Golf | Nature based activity | 1.8% |
| Concerts/music festivals | Cultural based activity | 7.0% |
| Buildings/castles/monuments | Cultural based activity | 28.0% |
| Attended a cultural event | Cultural based activity | 27.9% |
| Attended a sporting event | Cultural based activity | 2.0% |
| Theatre . | Cultural based activity | 2.7% |
| Family event (christening, wedding, etc.) | Cultural based activity | 2.7% |
| Museum | Cultural based activity | 28.7% |
| Zoo/amusement park | Cultural based activity | 6.3% |
| Sightseeing/excursions | Urban based activity | 36.2% |
| Training course/lecture/seminar | Urban based activity | 1.7% |
| Company visit | Urban based activity | 1.6% |
| Spa/wellness | Urban based activity | 3.6% |
| Disco/night club | Urban based activity | 4.2% |
| Restaurants/bars/cafés/snack kiosks | Urban based activity | 36.7% |
| Shopping | Urban based activity | 33.6% |
